# Free-Spin Dominated Magnetocaloric Effect in Dense Gd^3+^ Double Perovskites

**DOI:** 10.1021/acs.chemmater.2c00261

**Published:** 2022-03-29

**Authors:** EliseAnne C. Koskelo, Cheng Liu, Paromita Mukherjee, Nicola D. Kelly, Siân E. Dutton

**Affiliations:** Department of Physics, University of Cambridge, Cambridge CB3 0HE, United Kingdom

## Abstract

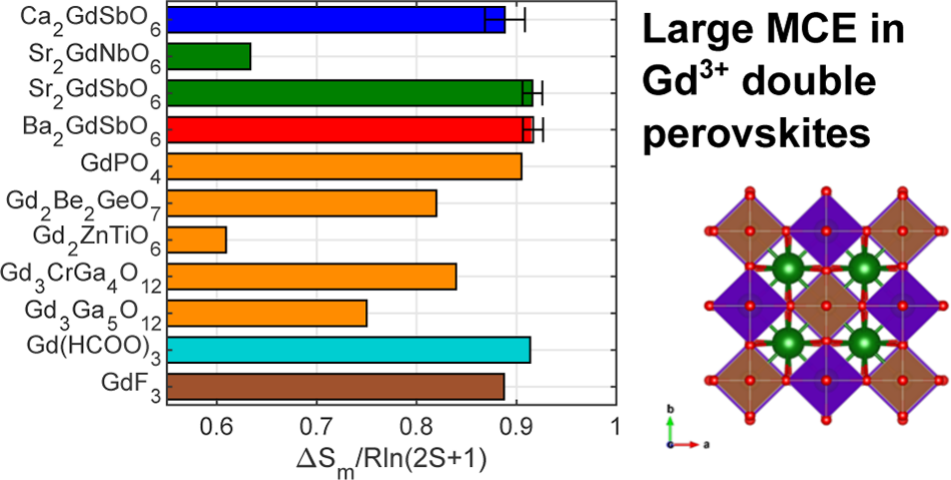

Frustrated lanthanide oxides with dense magnetic lattices are of fundamental interest for their potential in cryogenic refrigeration due to a large ground state entropy and suppressed ordering temperatures but can often be limited by short-range correlations. Here, we present examples of frustrated *fcc* oxides, Ba_2_GdSbO_6_ and Sr_2_GdSbO_6_, and the new site-disordered analogue Ca_2_GdSbO_6_ ([CaGd]_*A*_[CaSb]_*B*_O_6_), in which the magnetocaloric effect is influenced by minimal superexchange (*J*_1_ ∼ 10 mK). We report on the crystal structures using powder X-ray diffraction and the bulk magnetic properties through low-field susceptibility and isothermal magnetization measurements. The Gd compounds exhibit a magnetic entropy change of up to −15.8 J/K/mol_Gd_ in a field of 7 T at 2 K, a 20% excess compared to the value of −13.0 J/K/mol_Gd_ for a standard in magnetic refrigeration, Gd_3_Ga_5_O_12_. Heat capacity measurements indicate a lack of magnetic ordering down to 0.4 K for Ba_2_GdSbO_6_ and Sr_2_GdSbO_6_, suggesting cooling down through the liquid 4-He regime. A mean-field model is used to elucidate the role of primarily free-spin behavior in the magnetocaloric performance of these compounds in comparison to other top-performing Gd-based oxides. The chemical flexibility of the double perovskites raises the possibility of further enhancement of the magnetocaloric effect in the Gd^3+^*fcc* lattices.

## Introduction

Cryogenic cooling is imperative to modern technologies, including quantum computing and magnetic resonance imaging. While liquid He can be used to reach temperatures as low as 20 mK (using 3-He and 4-He) or 2 K (4-He only), it is a depleting resource, and sustainable alternatives capitalizing on magnetic, structural, and/or electric ordering of materials are of key interest.^1^ In adiabatic magnetic refrigerators (ADRs), an applied magnetic field induces a change in entropy of the spins of a material, Δ*S*_*m*_. When followed by adiabatic demagnetization, the system exhibits a proportional decrease in temperature Δ*T* as dictated by the magnetocaloric (MC) effect. MC materials are operable at temperatures above their ordering transition *T*_0_ and are often characterized by the maximum isothermal magnetic entropy change Δ*S*_*m*_ that can be achieved for a given change in field Δ*H*.^2^ Current top-performing MC materials are based on Gd^3+^ containing compounds, as the minimal single-ion anisotropy (*L* = 0) of the magnetic ions allows for full extraction of the theoretical entropy change in high magnetic fields.^3−5^ Recent advances in materials like Gd(HCOO)_3_, GdF_3_, and GdPO_4_, with dense magnetic sublattices, have highlighted the importance of weak magnetic correlations in enabling a large MC effect.^3,4,6^ However, these materials can be limited by a lack of tunability via chemical substitution and/or large volumes per magnetic ion.^5,6^ This limits the opportunities for tuning the magnitude and temperature of the maximum Δ*S*_*m*_.

On the other hand, frustrated magnetic oxides, in which the geometry of the lattice prevents all exchange interactions from being satisfied simultaneously, present a diverse class of MC candidates given their chemical stability and exotic magnetic properties such as a large ground state degeneracy and suppressed ordering temperature.^7^ Gd_3_Ga_5_O_12_, a frustrated garnet, is the standard among this class of materials, with an entropy change of −13.0 J/K/mol_Gd_ in a field of 7 T at 2 K.^5^ In addition to the garnet lattice which is comprised of two interpenetrating networks of bifurcating loops of ten corner-sharing *Ln*^3+^ triangles, a wealth of frustrated geometries exist, including the pyrochlore and kagome lattices and the *fcc* lattice, which as a set of edge-sharing tetrahedra is frustrated under antiferromagnetic exchange. However, one limitation of oxide materials is that their MC performance can be strongly influenced by short-range correlations.^8^ For example, Gd_3_Ga_5_O_12_ has a relatively large superexchange between Gd^3+^ ions, |*J*_1_| ∼ 100 mK,^9^ compared to |*J*_1_| ∼ 70 mK for GdF_3_.^3^

Lanthanide oxide double perovskites with the general formula *A*_2_*Ln*SbO_6_ (*A* = alkaline earth (Ba, Sr), *Ln* = lanthanide) represent a family of frustrated magnets in which *Ln* ions lie on an *fcc* magnetic sublattice, enforced by the rock-salt arrangement with the other *B* site cation, Sb^5+^. Chemical pressure via *A* site cation substitution can alter the distortion of a single *Ln*-ion tetrahedron due to small changes in the nearest neighbor *nn* distance dictated by rotations of the *B*O_6_ octahedra.^10^

Here we present *fcc* oxides with minimal superexchange interactions, up to 10 times smaller than those of other frustrated oxides. We report on the solid-state synthesis, structural characterization, bulk magnetic properties, and magnetocaloric effect in these materials, Ba_2_GdSbO_6_ and Sr_2_GdSbO_6_, and the new site-disordered Ca analogue, Ca_2_GdSbO_6_ ([CaGd]_*A*_[CaSb]_*B*_O_6_). We show that tuning of the *A* site ion influences the exchange through changes in the nearest neighbor distance and O–Gd–O bond angles, with small effect on the overall magnetocaloric performance, suggesting that free-spin behavior is dominant for 1.8 K and above. Furthermore, the low Curie–Weiss temperatures (∼0.8 K), frustrated lattice geometry, and lack of ordering of the Ba and Sr compounds suggest that cooling may persist through the cooperative paramagnetic regime to temperatures well below 0.4 K.

## Experimental Methods

### Solid State Synthesis

Powder samples, of ∼1 g, of Ba_2_GdSbO_6_, Sr_2_GdSbO_6_, and Ca_2_GdSbO_6_ were prepared as described in the literature.^10^ Stoichiometric mixtures of predried gadolinium(III) oxide (99.999%, Alfa Aesar REacton), antimony(V) oxide (99.9998%, Alfa Aesar Puratronic), and the appropriate alkaline earth carbonate, barium carbonate (99.997%, Alfa Aesar Puratronic), strontium carbonate (99.99%, Alfa Aesar), or calcium carbonate (99.99%, Alfa Aesar Puratronic) were initially ground using a mortar and pestle and heated in air at 1400 °C for 24 h. Heating was repeated until the amount of impurity phases as determined by X-ray diffraction no longer reduced upon heating (one additional 24 h cycle). Ba_2_GdSbO_6_, Sr_2_GdSbO_6_, and Ca_2_GdSbO_6_ each contain impurity phases of Gd_3_SbO_7_ of 0.45(4), 0.67(4), and 0.48(1) wt %, respectively, with antiferromagnetic ordering at 2.6 K.^11^

### Structural Characterization

Room temperature powder X-ray diffraction (XRD) measurements were carried out using a Bruker D8 Advance diffractometer (Cu Kα radiation, λ = 1.54 Å). Data was collected with *d*(2θ) = 0.01° from 2θ = 15–150°, with an overall collection time of 2–3 h. During each scan, the sample stage was rotated to avoid preferred orientation effects. Additional high resolution X-ray powder diffraction measurements were conducted at the I11 beamline at the Diamond Light Source using a position sensitive detector for Ca_2_GdSbO_6_ at room temperature and at 100 K. Data was collected with λ = 0.826866 Å from 2θ = 8–100° using a position sensitive detector, with an overall collection time of 1 min. The powder sample was mounted in a 0.28 mm diameter capillary inside a brass sample holder.

Rietveld refinements^12^ of the powder XRD data were completed using the Diffrac.Suite TOPAS5 program.^13^ Peak shapes were modeled using a pseudo-Voigt function,^14^ and the background was fit using a 13-term Chebyshev polynomial. Except for Ca_2_GdSbO_6_, where synchrotron XRD data was available, all Debye–Waller factors were kept constant at the literature reported values from powder neutron diffraction.^10,15^ A cylindrical correction was used to correct for capillary absorption in the I11 data as well as a Lorentzian/Gaussian model to account for strain broadening effects on the peak shape.^16^

### Magnetic Characterization

Magnetic susceptibility χ(*T*) = d*M*/d*H* (∼ *M*/*H* in the low field limit) and isothermal magnetization *M*(*H*) measurements were conducted using a Quantum Design Magnetic Properties Measurement System (MPMS) with a superconducting interference device (SQUID) magnetometer. Susceptibility measurements were made in zero-field-cooled conditions (μ_0_*H* = 1000 Oe, where *M*(*H*) is linear and the χ = *M*/*H* approximation is valid) over a temperature range of 1.8–300 K and in field-cooled conditions from *T* = 1.8–30 K. *M*(*H*) measurements were made over a field range of 0 ≤ μ_0_*H* ≤ 7 T, in steps of 0.2 T from 2 to 20 K, in 2 K steps from 2 to 10 K, and in 5 K steps from 10 to 20 K. The magnetic entropy change for a field *H*_max_ relative to zero field was extracted from *M*(*H*) by computing the temperature derivative of the magnetization using
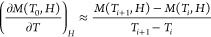
1and then integrating in discrete steps of 0.1 T across fields using the trapezoidal method:
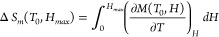
2

### Low Temperature Heat Capacity

Magnetic heat capacity measurements were carried out using a Quantum Design PPMS using the 3-He probe (0.4 ≤ *T* ≤ 30 K) in zero field. Equal masses of the sample and Ag powders were mixed with a mortar and pestle and pressed into a 0.5 mm pellet to enhance the thermal conductivity. Pellets were mounted onto the sample platform using *N*-grease to ensure thermal contact. Addenda measurements of the sample platform and grease were calibrated at each temperature before measurement. The sample heat capacity, *C*_*p*_, was obtained from the measured heat capacity, *C*_*tot*_, by subtracting the Ag contribution using the literature values.^17^ The magnetic heat capacity *C*_*mag*_ was obtained from a subtraction of the lattice contribution *C*_*lat*_ from the sample heat capacity *C*_*p*_:
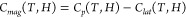
3The lattice contribution *C*_*lat*_ was determined using least-squares fits of the zero-field *C*_*p*_ at high temperatures (8–50 K) to the Debye model:
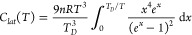
4where *T*_*D*_ is the Debye temperature, *R* is the molar gas constant, and *n* is the number of atoms per formula unit. The total magnetic entropy, relative to the lowest temperature *T*_*i*_ measured, was computed using
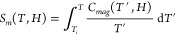
5numerically for the temperatures measured.

## Structural Characterization

**Figure 1:**
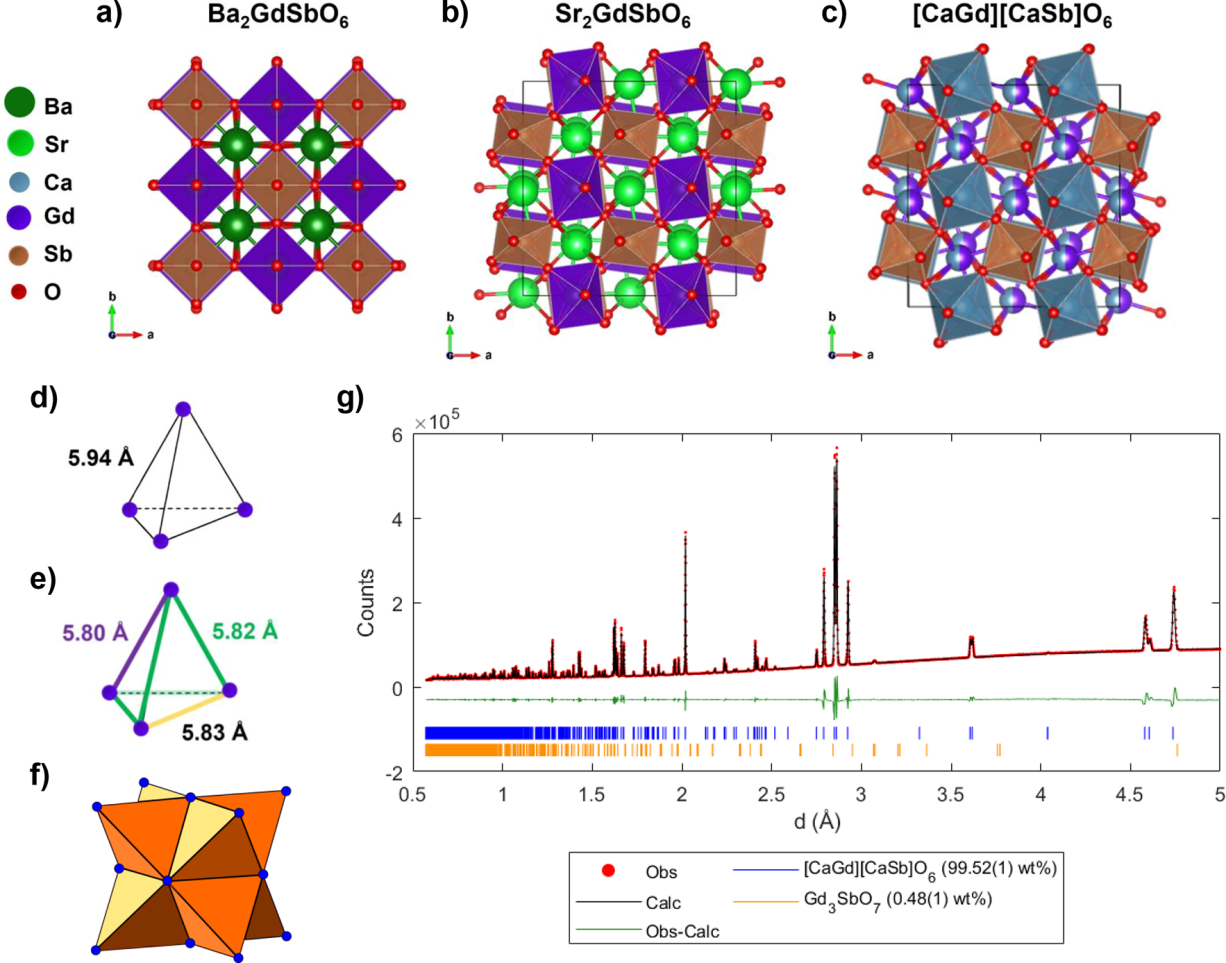
Crystal structures of the double perovskites (a) Ba_2_GdSbO_6_, (b) Sr_2_GdSbO_6_, and (c) [CaGd]_*A*_[CaSb]_*B*_O_6_. In [CaGd]_*A*_[CaSb]_*B*_O_6_, the Gd^3+^ ions lie in a disordered arrangement with Ca^2+^ on the *A* sites. The rock-salt ordering of Gd^3+^ and Sb^5+^ on the *B* sites produce a *fcc* magnetic sublattice, which is (d) uniform for Ba_2_GdSbO_6_ and (e) distorted for Sr_2_GdSbO_6_, with the listed side lengths. (f) The *fcc* sublattice is a frustrated geometry because it composes a network of edge-sharing tetrahedra. (g) High-resolution powder X-ray diffraction Rietveld refinement of [CaGd]_*A*_[CaSb]_*B*_O_6_. Observed intensities and calculated intensities obtained from a Rietveld refinement are shown as red circles and a black line, respectively; the difference (data – fit) is shown by a green line. Reflection positions are indicated by blue and orange tick marks, for phases [CaGd]_*A*_[CaSb]_*B*_O_6_ and the small phase impurity Gd_3_SbO_7_ (0.48(1)% by weight), respectively.

Powder X-ray diffraction indicates formation of an almost phase pure sample for *A*_2_GdSbO_6_ (*A* = {Ba, Sr, Ca}). Rietveld refinements, Figure 1 and Table 1, show that all three compounds exhibit small 0.7 wt % impurities of Gd_3_SbO_7_.^11^ The structures of Ba_2_GdSbO_6_ and Sr_2_GdSbO_6_ are consistent with those of prior reports.^10^ Both materials exhibit full rock-salt ordering of Gd^3+^ and Sb^5+^ on the *B* sites, attributable to the large charge and ionic radii differences between cations; Ba_2_GdSbO_6_ forms a cubic  structure, resulting in a uniform tetrahedron of Gd^3+^ ions, while Sr_2_GdSbO_6_ forms a monoclinic *P*2_1_/*n* structure, resulting in a distorted tetrahedron of Gd^3+^ ions, Figure 1.^10^

**Table 1:** Lattice Parameters and Crystal Structures of Ba_2_GdSbO_6_, Sr_2_GdSbO_6_, and Ca_2_GdSbO_6_ ([CaGd]_*A*_[CaSb]_*B*_O_6_) as Determined from Rietveld Refinements of Powder X-ray Diffraction at Room Temperature for Ba_2_GdSbO_6_ and Sr_2_GdSbO_6_ and at 100 K for [CaGd]_*A*_[CaSb]_*B*_O_6_^a^

atom	Wyckoff position	*x*	*y*	*z*	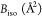
Ba_2_GdSbO_6_, *Fm*3̅*m*					
Ba	8*c*	0.25	0.25	0.25	0.67
Gd	4*a*	0	0	0	0.48
Sb	4*b*	0.5	0.5	0.5	0.41
O	24*e*	0.257(2)	0	0	0.76
*a* (Å)	8.47517(2)				
Gd_3_SbO_7_ (wt %)	0.45(4)				
χ^2^	1.41				
*R*_*wp*_	11.6				
Sr_2_GdSbO_6_, *P*2_1_/*n*					
Sr	4*e*	0.0105(5)	0.0346(2)	0.2489(7)	0.79
Gd	2*d*	0.5	0	0	0.24
Sb	2*c*	0	0.5	0	0.39
O(1)	4*e*	0.253(3)	0.317(3)	0.021(3)	0.79
O(2)	4*e*	0.190(4)	0.761(3)	0.042(3)	0.79
O(3)	4*e*	–0.086(2)	0.486(2)	0.239(3)	0.79
*a* (Å)	5.84113(5)				
*b* (Å)	5.89402(5)				
*c* (Å)	8.29127(7)				
β (deg)	90.2373(7)				
Gd_3_SbO_7_ (wt %)	0.67(4)				
χ^2^	1.20				
*R*_*wp*_	8.7				
[CaGd]_*A*_[CaSb]_*B*_O_6_, *P*2_1_/*n*					
Ca_1_/Gd	4*e*	–0.0174(1)	0.05939(7)	0.25403(8)	0.90(1)
Ca_2_	2*d*	0.5	0	0	0.68(8)
Sb	2*c*	0	0.5	0	0.54(1)
O(1)	4*e*	0.1660(8)	0.2149(7)	–0.0728(6)	1.25(5)
O(2)	4*e*	0.2089(8)	0.1769(7)	0.5511(6)	1.25(5)
O(3)	4*e*	1.1205(7)	0.4390(7)	0.2254(5)	1.25(5)
*a* (Å)	5.58025(2)				
*b* (Å)	5.84820(2)				
*c* (Å)	8.07706(2)				
β (deg)	90.3253(2)				
OccCa1	0.5				
Occ_Gd_	0.5				
Gd_3_SbO_7_ (wt %)	0.48(1)				
χ^2^	5.80				
*R*_*wp*_	3.7				

a The Debye–Waller factors for Ba_2_GdSbO_6_ and Sr_2_GdSbO_6_ were kept constant to values reported in the literature for the related compounds Ba_2_DySbO_6_ and Sr_2_GdSbO_6_, respectively.^10,15^.

Structural refinements, Table 1, indicate that Ca_2_GdSbO_6_ adopts the monoclinic space group *P*2_1_/*n*. However, additional reflections ((011) and (101)) at 2θ ≈ 18° (*d* = 4.6 – 4.8 Å) indicate that Gd^3+^ occupies the *A* sites as in the case of its of nonmagnetic analogue [CaLa]_*A*_[CaSb]_*B*_O_6_.^18^ Refinement of the occupancy of Gd^3+^ and Ca^2+^ across the *A* and *B* sites indicated that Gd^3+^ only occupies the *A* sites, and so this cation distribution was fixed in subsequent refinements. Thus, a clearer description of the compound is [CaGd]_*A*_[CaSb]_*B*_O_6_. (Here, the bracketed notation [*XY*]_*A*_[*LZ*]_*B*_O_6_ refers to a double perovskite in which species *X* and *Y* lie on the *A* sites and species *L* and *Z* lie on the *B* sites.) Consistent with a prior study of Mn-doped [Ca_1–*x*_Sr_*x*_Gd]_*A*_[CaSb_1–*y*_]_*B*_O_6_:Mn_*y*_ (*x* = 0.4, *y* = 0.003)^19^ and the structure of [CaLa]_*A*_[CaSb]_*B*_O_6_,^18^ we find a disordered arrangement on the *A* sites with half of the sites occupied by the Gd^3+^ ions and the other half by Ca^2+^ and rock-salt ordering of Ca^2+^ and Sb^5^ on the *B* sites.

There is no evidence of *A*-site ordering in the compound with no further superstructure peaks observed in the synchrotron XRD. This is likely due to the minimal charge and ionic radii differences of the Ca^2+^ and Gd^3+^ ions which rules out rock-salt and columnar ordering on the *A* sites.^20^

To analyze whether the site disorder in [CaGd]_*A*_[CaSb]_*B*_O_6_ is due to close-packing efficiency considerations, we computed the Goldschmidt tolerance factor (GTF), *t*. *t* predicts whether the ionic radii of the *A*-site cation and *B*-site cation are well-scaled for a cubic structure in which *A*-site cations lie at the cavities of *B*–O octahedra.^21^ It can be extended to double perovskites with the formula *AA*′*BB*′O_6_ by computing average *A*-site and *B*-site ionic radii so that
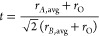
6where .^22^ The GTF for [CaCa]_*A*_[GdSb]_*B*_O_6_ in which Gd^3+^ and Sb^5+^ are located on the *B* sites is 0.82 compared to 0.80 for [CaGd]_*A*_[CaSb]_*B*_O_6_ in which Gd^3+^ lies on the *A* sites. Since *t* ≈ 1 in stable perovskite structures, these results suggest that the Gd^3+^ ions should lie preferentially on the *B*-site.

It is also possible that charge differences of the cations stabilize the observed disordered structure.^20^ However, an additional calculation using a charge-based tolerance factor also predicted [CaCa]_*A*_[GdSb]_*B*_O_6_ as the more stable structure.^23^ Thus, the *A* site occupancy of Gd^3+^ is unlikely to be due to the greater close cubic packing efficiency or charge differences between cations. It could be that the additional entropy associated with a random distribution of Ca^2+^ and Gd^3+^ on the *A* site favors the observed cation distribution, although we note that a random distribution of Ca^2+^/Gd^3+^ across both sites is more entropically favorable. Changes to the synthesis procedure, e.g., slow cooling, may result in differences in the Ca^2+^/Gd^3+^ distribution but are beyond the scope of this study.

## Magnetic Characterization and Results

**Figure 2:**
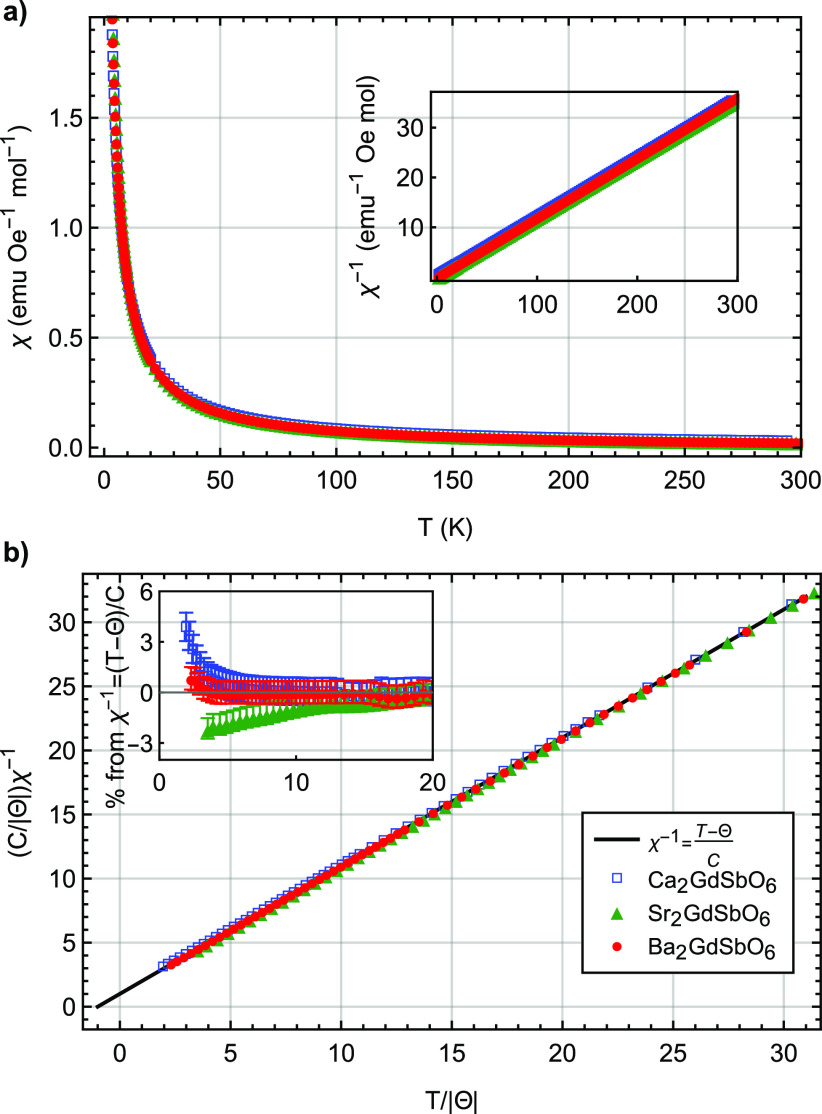
(a) Low-field magnetic susceptibility χ ≈ *M*/*H* versus temperature *T* of *A*_2_GdSbO_6_ (*A* = {Ba, Sr, Ca}) (inset: χ^–1^ versus *T*). (b) Dimensionless inverse magnetic susceptibility scaled by the appropriate factors of the Curie constant *C* and Curie–Weiss temperature Θ for each material. The error bars are smaller than the points in the graph. All compounds remain paramagnetic down to 1.8 K. The inset depicts the percent deviation of χ^–1^ from the Curie–Weiss law, *C*/(*T* – Θ). Error bars are determined assuming a 0.1 mg mass error.

The zero-field-cooled (ZFC) magnetic susceptibility χ(*T*), Figure 2, indicates paramagnetic behavior of each material down to 1.8 K, in agreement with prior investigations.^10^ Curie–Weiss fits to the inverse susceptibility χ^–1^(*T*) were conducted from temperatures *T* = 8 to 50 K. For all compositions, the negative Curie–Weiss temperatures Θ_*CW*_, Table 2, indicate antiferromagnetic interactions between spins that increase in strength from the monoclinic (*A* = Sr) to cubic lattice (*A* = Ba). The Curie–Weiss law can be written in a dimensionless form given by  (for Θ_*CW*_ < 0), where Θ_*CW*_ is the Curie temperature and *C* is the Curie constant.^24^ This dimensionless form can elucidate the presence of short-range correlations from the inverse magnetic susceptibility and enable a comparison across compounds.^24,25^ In these dimensionless units, free-spin behavior is indicated by the linear relationship of  with  with a *y*-intercept of 1. Positive (negative) deviations from linearity can indicate antiferromagnetic (ferromagnetic) short-range correlations between spins. Figure 2 highlights the minimal short-range correlations of each *A*_2_GdSbO_6_ compound, all of which exhibit extremely small deviations of less than 5% from free-spin behavior at 1.8 K. As a comparison, the MgCr_2_O_4_ spinels, which order at , exhibit 20–60% deviations at temperatures of .^25^ The positive deviations of [CaGd]_*A*_[CaSb]_*B*_O_6_ are likely to correspond to antiferromagnetic short-range correlations rather than disorder-induced quantum fluctuations. Field-cooled measurements below 20 K indicate no hysteresis (see Figures S1 and S2).

**Table 2:** Fit Nearest Neighbor Exchange *J*_1_ for the *fcc* Ba_2_GdSbO_6_ and Sr_2_GdSbO_6_ and Overall Exchange Field, *a*_*ex*_*z*, for Site Disordered Ca_2_GdSbO_6_ Based on Curie–Weiss Analysis of the Inverse Magnetic Susceptibility χ^–1^ (Equation S1) and a Mean-Field Model Fit (Equations 7 and 8) to the Isothermal Magnetization Curves from 2 to 20 K^a^

	from χ^–1^ fit		from *M*(*H*) fit	
	Θ (K)	(K)	*J*_1_ (K)	*R*^2^
Ba_2_GdSbO_6_	–0.78(1)	0.0124(2)	0.0113(5)	1.0000
Sr_2_GdSbO_6_	–0.51(1)	0.0081(2)	0.0070(2)	1.0000

	Θ (K)	(T)	*za*_*ex*_ (T)	*R*^2^
Ca_2_GdSbO_6_	–0.92(1)	–0.0652(5)	–0.0617(7)	0.9999

a Quoted uncertainties represent a 95% confidence interval from the least squares fits.

A mean-field estimate for the nearest neighbor (*nn*) superexchange *J*_1_, Table 2, indicates weak coupling between spins in the *fcc* compounds, ∼10 mK, compared to 100 mK in Gd_3_Ga_5_O_12_.^9^ Although the number of nearest neighbors is not constant for the disordered [CaGd]_*A*_[CaSb]_*B*_ analogue, a mean-field estimate for the six nearest *A* sites (*R*_*nn*,*avg*_ = 4.063(3) Å from distances 2 × 3.918(3), 2 × 4.171(3), 4.162(3), and 4.038(3) Å) was computed, giving an order of magnitude estimate for *J*_1_.

**Figure 3:**
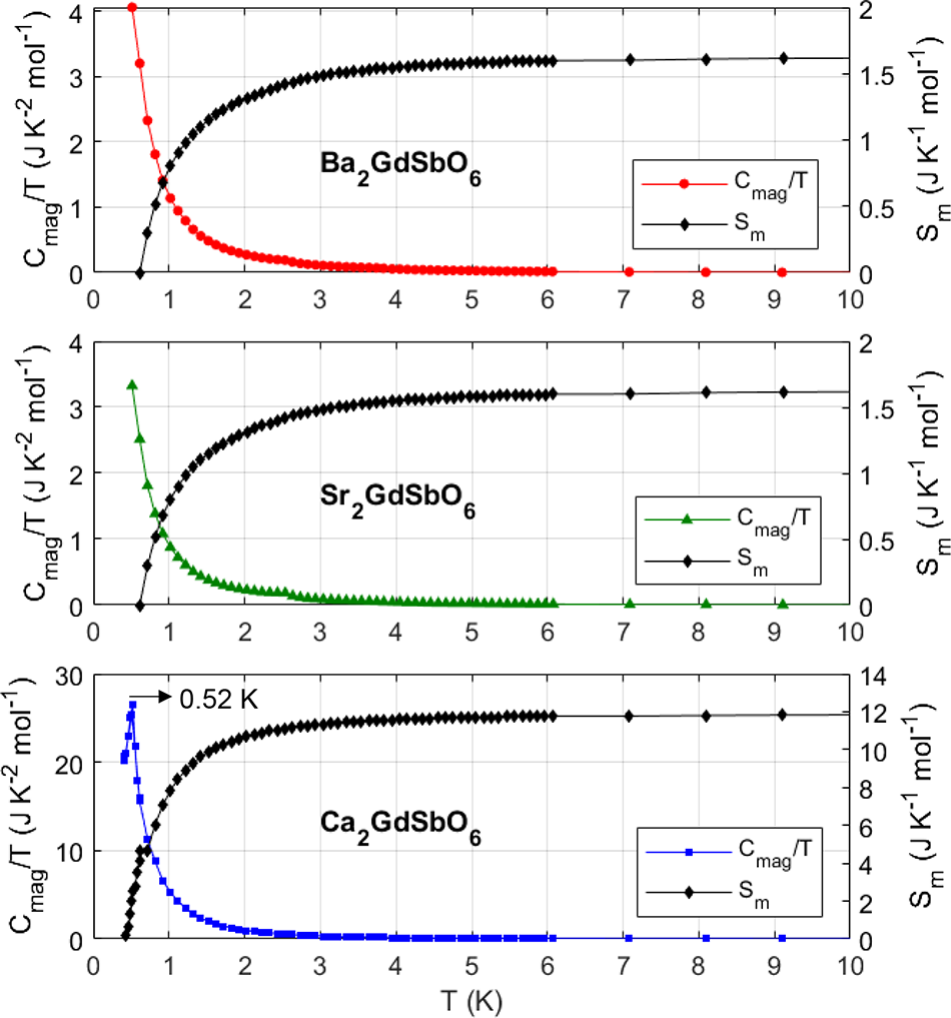
Zero field magnetic heat capacity normalized by temperature *C*_*mag*_/*T* of *A*_2_GdSbO_6_ (*A* = {Ba, Sr, Ca}) and corresponding magnetic entropy released from 0.4 to 10 K. The Debye temperatures for each material (Table 3) were found from fits of the total heat capacity to the Debye model (eq 4) from 6 to 30 K. The small anomaly in each measurement at 2.6 K is likely due to the ordering of the ∼0.5–1 wt % Gd_3_SbO_7_ impurity.^11^

The zero field measured magnetic heat capacities *C*_*mag*_ of *A*_2_GdSbO_6_ (*A* = {Ba, Sr, Ca}) from 0.4 to 30 K are depicted in Figure 3. The Debye temperatures *T*_*D*_ were determined from fits to the Debye model (eq 4) from 6 to 30 K, Table 3. No magnetic ordering is observed in the *fcc* compounds down to 0.4 K. The magnetic entropy of Ba_2_GdSbO_6_ and Sr_2_GdSbO_6_ at 10 K, relative to that at 0.4 K, is 1.6 J/K/mol_Gd_, corresponding to only 10% of that available for *S* = 7/2 Heisenberg spins. In contrast, the site-disordered Ca_2_GdSbO_6_ exhibits a sharp λ-type anomaly at 0.52 K, indicative of a long-range ordering transition. The total entropy contained from 0.4 to 30 K is 12 J/K/mol_Gd_, or 0.7*R* ln(2*S* + 1). This ordering transition may be due to the larger dipolar interaction *D* in Ca_2_GdSbO_6_, Table 3, which is  times that of Ba_2_GdSbO_6_ and Sr_2_GdSbO_6_, and/or a different Gd^3+^ ion arrangement.

**Table 3:** Curie–Weiss Temperature Θ, Ordering Temperature *T*_0_, Debye Temperature *T*_*D*_, and Corresponding Estimates for the Mean-Field *nn* Exchange *J*_1_ and Dipolar Interaction *D* for *A*_2_GdSbO_6_ (*A* = {Ba, Sr, Ca}) and Reported Top-Performing Gd-Based Magnetocaloric Materials Compared to Three Commonly Used Paramagnetic Salts: Ferric Ammonium Alum (FAS), Copper Ammonium Sulfate (CAS), and Copper Potassium Sulfate (CPS).^a^^,^^b^

	Θ_*CW*_ (K)	*J*_1_ (K)	*D* (K)	*D*/*J*_1_	*T*_0_ (K)	*T*_*D*_ (K)
Ba_2_GdSbO_6_	–0.78(1)	0.0124(2)	0.0116	0.94	<0.4	365
Sr_2_GdSbO_6_	–0.51(1)	0.0081(2)	0.0123	1.5	<0.4	475
Ca_2_GdSbO_6_ (*z* ≈ 6)	–0.92(1)	0.029	0.037	1.3	0.52	360
Sr_2_GdNbO_6_^31^	3.2	–0.051	0.012	–0.24	∼2	-
Gd(HCOO)_3_^3^	–0.3	0.0286	0.0393	1.4	0.8	168
GdPO_4_^4^	–0.9	0.029	0.0362	1.3	0.8	220
GdF_3_^5^	+0.7	–0.067	0.0503	–0.75	1.25	284(3)
Gd_3_Ga_5_O_12_^9,33,34^	–2.6(1)	0.107	0.0457	0.43	≈0.14	≈500
Gd_2_ZnTiO_6_^27^	–4.0	0.024	0.044	1.84	2.43	156.4
Gd_2_Be_2_GeO_7_^29^	–4.09(5)	0.156(2)	0.043	0.28	<2	-
FAA^35−37^	0.042	–0.007	0.010	–1.4	0.026	80
CAS^38,39^	0.010(5)	–0.007(3)	0.0070	–1.1	-	-
CPS^38,39^	0.016(5)	–0.010(3)	0.0070	–0.7	-	-

a The mean field *nn* exchange was calculated using Equation S1 and the dipolar interaction using *D* = *D*_*nn*_/*S*(*S* + 1) (with *D*_*nn*_ from Equation S2 as in refs (9 and 32). The *nn* exchange estimated from the Θ reported for Gd(HCOO)_3_ and GdF_3_ was taken to be along the Gd–Gd chains, so that *z* = 2, and in the Gd–Gd planes for Gd_2_Be_2_GeO_7_, so that *z* = 5. Gd_2_ZnTiO_6_ was treated as having 6 *nn* with an average distance of 3.83 Å. Ca_2_GdSbO_6_ was treated as having *z* = 6 with an average distance of 4.063(3) Å, as described in the text. CAS and CPS were treated as having 6 *nn* with an average distance of 7.1 Å as in ref (38) and FAA as having 2 *nn* at 6.24 Å.

b The reports of magnetism in Gd_3_Ga_5_O_12_ are highly sample dependent.^40,41^

**Figure 4:**
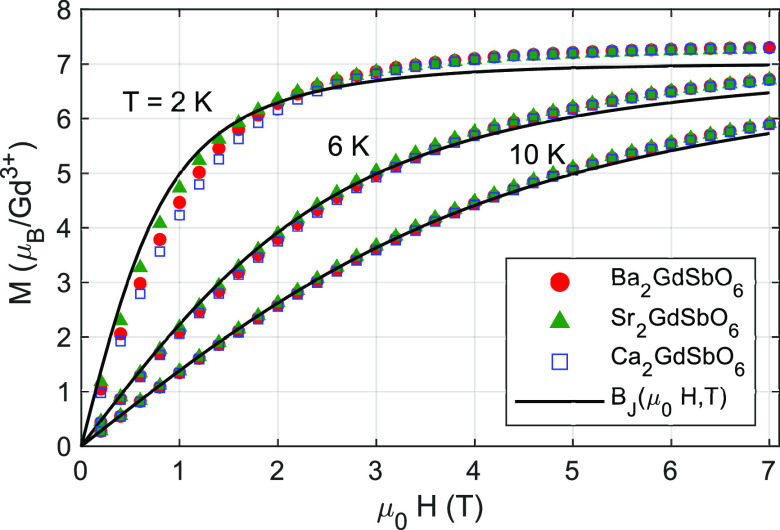
Isothermal magnetization of of *A*_2_GdSbO_6_, *A* = {Ca,Sr,Ba} at *T* = 2, 6, and 10 K compared to the Brillouin function for free *S* = 7/2 spins. Error bars are smaller than the data points.

**Figure 5:**
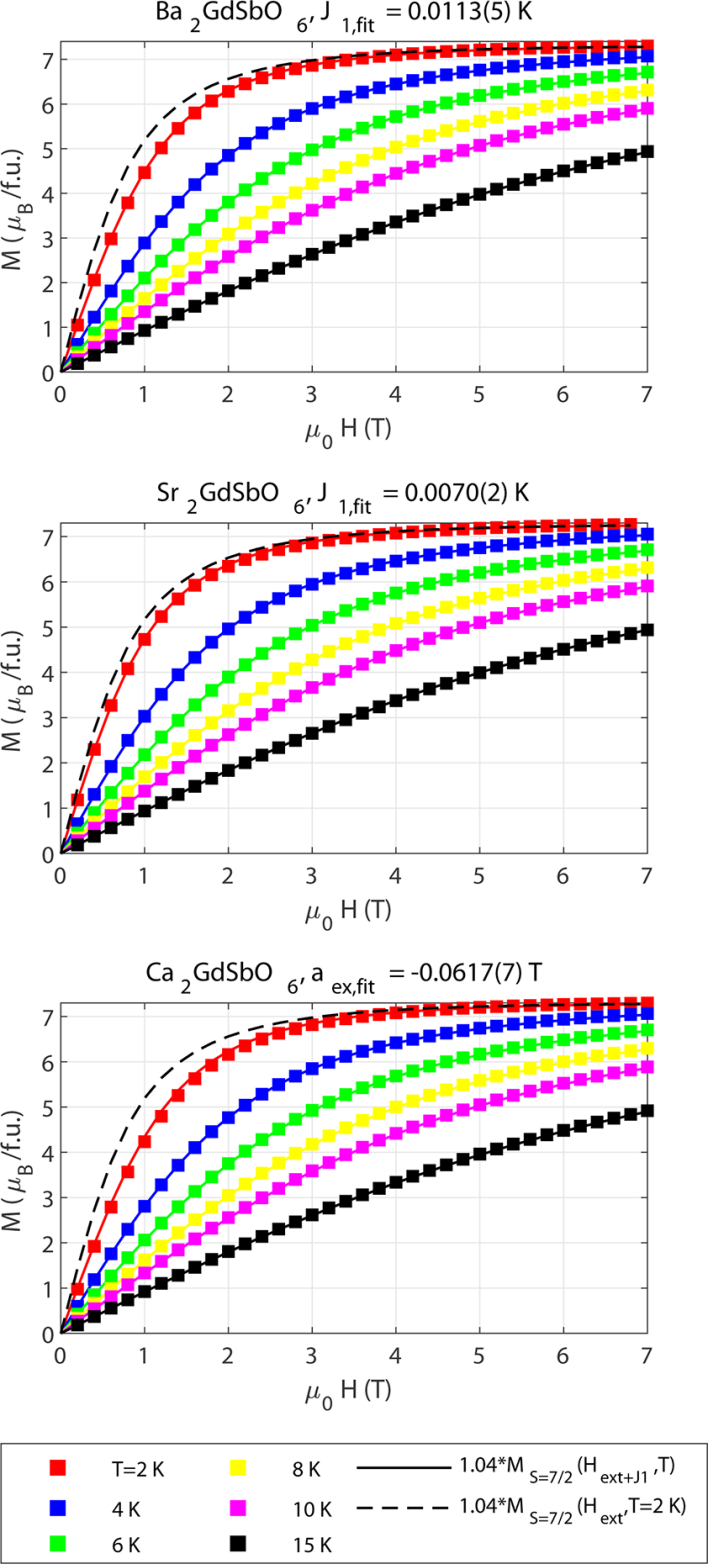
Isothermal magnetization *M* versus field μ_0_*H* for *A*_2_*Ln*SbO_6_ (*A* = {Ba, Sr, Ca}). Measured data are shown as points, while theoretical predictions based on a fit of the *nn* exchange, *J*_1_, for each material are shown as solid lines. The prediction of free Heisenberg spins at 2 K is shown as a dashed line. Ca_2_GdSbO_6_ was fit to an overall exchange field, *a*_*ex*_, with the number of *nn*, *z*, set to one, due to the presence of site disorder. All error bars are smaller than the data points.

**Figure 6:**
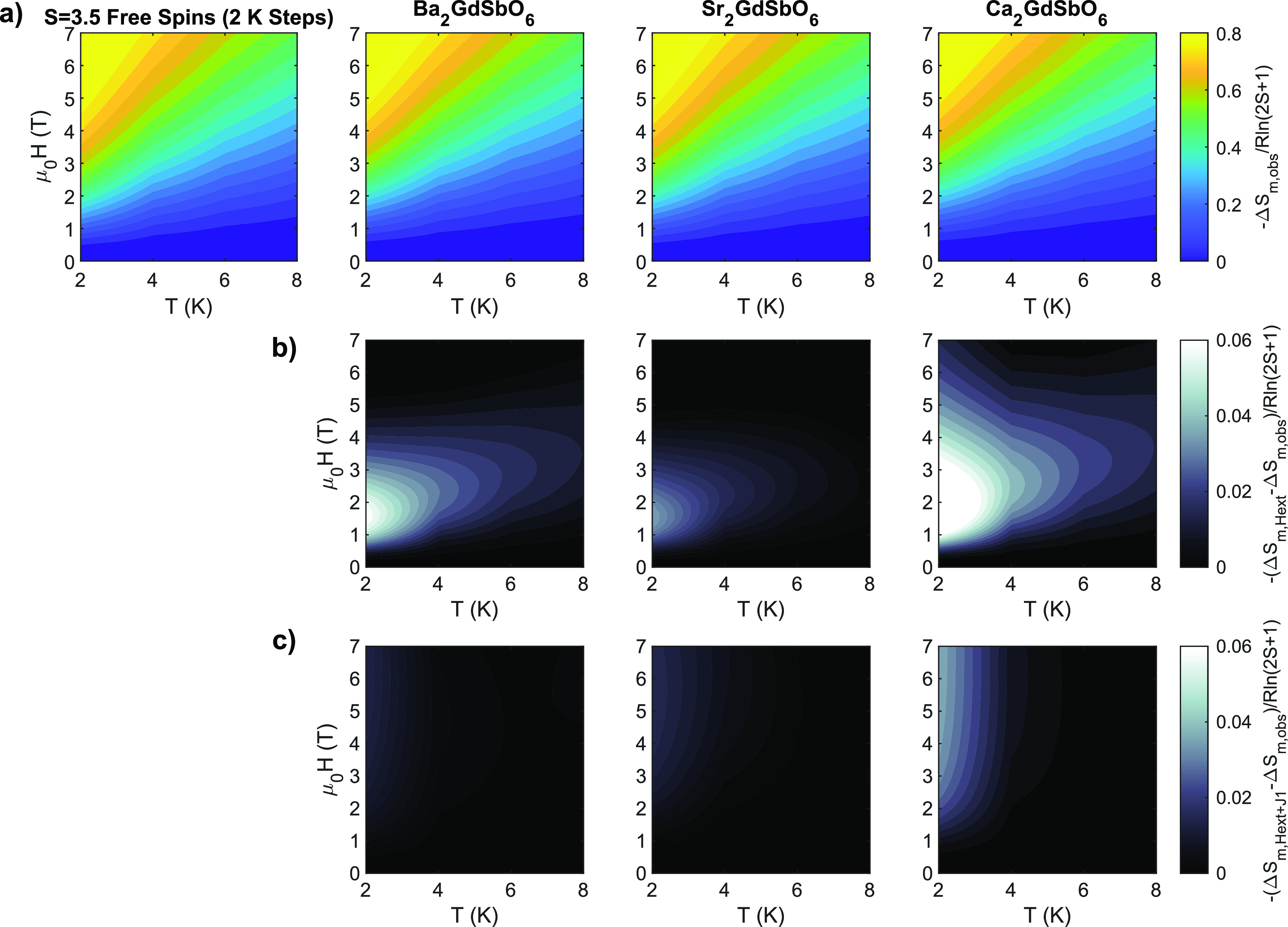
(a) Magnetic entropy change Δ*S*_*m*_ for uncoupled *S* = 7/2 spins determined from the Brillouin function *M*_*S*=7/2_(*H*, *T*) with Δ*T* = 2 K and Δ*H* = 0.1 T steps, compared to the measured Δ*S*_*m*,*obs*_ for the three *A*_2_*Ln*SbO_6_ compounds, *A* = {Ba, Sr, Ca}. (b) Differences between the theoretical entropy change for free-spins, Δ*S*_*m*,*Hext*_, and that measured for *A*_2_*Ln*SbO_6_, Δ*S*_*m*,*obs*_. All compounds exhibit deviations of 0.04*R* ln(2*S* + 1) to 0.08*R* ln(2*S* + 1) at low (1–2 T) fields, possibly indicating the contribution of AFM superexchange. (c) Differences between the theoretical magnetic entropy change predicted for the *nn* exchange field model using the fit *J*_1_, Δ*S*_*m*,*Hext*+*J*1_, and the measured data, Δ*S*_*m*,*obs*_, for *A*_2_*Ln*SbO_6_. Using this *nn* exchange field model, the deviations are reduced to less than 0.01*R* ln(2*S* + 1) for Ba_2_GdSbO_6_ and Sr_2_GdSbO_6_ and to 0.04*R* ln(2*S* + 1) for Ca_2_GdSbO_6_.

Isothermal magnetization *M*(*H*) measurements, shown in Figures 4 and 5, were conducted to measure the magnetocaloric effect, Δ*S*_*m*_(*H*, *T*). At 2 K, all compounds are saturated by μ_0_*H* = 7 T at the maximum value for free Heisenberg spins, *g*_*J*_*J* = 7 μ_*B*_/Gd^3+^. The measured magnetic entropy change Δ*S*_*m*,*obs*_ for applied fields of 0.2–7 T at temperatures of 2–8 K is shown in Figure 6. The *A*_2_GdSbO_6_ compounds are a high performing set of dense lanthanide oxides, reaching above 90% of the maximum entropy change (per mol Gd) predicted for uncoupled Heisenberg spins, *R* ln(2*S* + 1), in a 7 T field at 2 K. Somewhat surprisingly, the presence of site disorder and magnetic ordering at 0.52 K in [CaGd]_*A*_[CaSb]_*B*_O_6_ has only a small effect on the overall magnetocaloric performance in this temperature regime, suggesting that minimal superexchange may play a role in enhancing the magnetocaloric effect in the liquid He regime.

## Investigating the Role of Superexchange on the High Magnetocaloric Effect

To elucidate the origin of the large magnetocaloric effect in these materials, we investigate two models: first, an uncoupled model of *S* = 7/2 Heisenberg spins, and second, a mean-field model that accounts for antiferromagnetic superexchange between Gd^3+^ ions.

### Uncoupled Spin Analysis

Predictions for the theoretical magnetic entropy change Δ*S*_*m*,*Hext*_ of uncoupled Gd^3+^ (*S* = 7/2) spins were computed from the isothermal magnetization curves determined from the Brillouin function and maximum saturation *g*_*J*_*J* (eq S4). *M*(*H*, *T*) curves were evaluated at 2–10 K, with 2 K steps, and from 0 to 7 T, with 0.1 T steps, in accordance with the measured temperatures and fields. Figure 6a,b demonstrates that the predictions, Δ*S*_*m*,*Hext*_, for paramagnetic *S* = 7/2 spins are remarkably close to the measured entropy changes, Δ*S*_*m*,*obs*_, for the three *A*_2_GdSbO_6_ compounds. Ba_2_GdSbO_6_ and Ca_2_GdSbO_6_ exhibit a maximum deviation of 0.09*R* ln(2*S* + 1), corresponding to 10% of the maximum entropy change of 0.9*R* ln(2*S* + 1) predicted for free-spins. These deviations occur at low fields (1–3 T) and smaller temperatures (∼2–4 K), in accordance with small antiferromagnetic exchange indicated in the Curie–Weiss analysis. The deviations of Δ*S*_*m*_ from Δ*S*_*m*,*S*=7/2_ for Sr_2_GdSbO_6_ are lower, only 0.04*R* ln(2*S* + 1), and are concentrated at 2 K. None of the measured compounds exceeds the magnetic entropy change predicted for free *S* = 7/2 spins; this result is in agreement with the ∼1 K Curie–Weiss temperatures of the materials which imply that, for the measured 2–10 K temperatures, the materials are paramagnetic.

### Incorporating an Exchange Field

A recent paper on the kagome compound Gd_3_Mg_2_Sb_3_O_14_ showed that an *nn* exchange field can be used to explain deviations from free-spin behavior below the saturation field.^26^ Here, we apply this model to characterize the *nn* exchange *J*_1_ in *A*_2_GdSbO_6_ (*A* = {Ba, Sr, Ca}) and its role in the isothermal field gradient of the entropy, (*∂S*/*∂H*)_*T*_ = (*∂M*/*∂T*)_*H*_, the determining factor in the magnetocaloric effect.

The model treats antiferromagnetic coupling between *S* = 7/2 spins using a mean-field approach so that the net field experienced by a single spin, *S*_*i*_, is composed of the external field *H*_*ext*_ and the exchange field *H*_*exc*_ due to *z* nearest neighbors, which scales with the bulk magnetization of the system.^26^ This mean-field approach is justified for *A*_2_GdSbO_6_ because the Curie–Weiss analysis indicates that the compounds are paramagnetic in the given temperature range (2–22 K) and thus that the role of quantum fluctuations need not be considered.

Since *L* = 0 for Gd^3+^, the exchange constant *J*_1_ can be assumed to be isotropic, so that the exchange field is given by
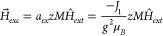
7where *M* is the bulk magnetization in units of the Bohr magneton and *a*_*ex*_ is the “field parameter” in units of magnetic field.^26^ The bulk magnetization of the system at a given temperature *T* and external field *H*_*ext*_ is given by the roots of the transcendental equation:
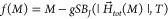
8where *B*_*J*_ is the Brillouin function (eq S4).^26^

Using this model, estimates of the nearest neighbor exchange *J*_1_ in Ba_2_GdSbO_6_ and Sr_2_GdSbO_6_ and overall exchange field *a*_*ex*_ in Ca_2_GdSbO_6_ were found using least-squares fits of the observed isothermal magnetization curves *M*(*H*) at 2–20 K, Figure 5. The free-spin magnetization  was used as an initial parameter for the mean-field exchange model magnetization (eq 8). For the *fcc* A = {Ba, Sr}, the number of *nn*, *z*, was set to 12, while the site disordered Ca version was fit to an overall exchange field, *a*_*ex*_, with *z* = 1. All compounds were fit with a scaled fraction of *M*_*sat*_ = *gS*; here, *M*_*sat*_ = 1.04*gSμ*_*B*_, the observed saturated value of the magnetization.

Table 2 shows the fit *nn* exchange interaction *J*_1_ and overall exchange field *a*_*ex*_ for each of the materials, compared to estimates from low field susceptibility measurements. Overall, there is broad agreement across the two methods. The Curie–Weiss superexchange estimates are slightly larger than from the *M*(*H*) curves, which could be due to a small contribution from thermally excited states at the higher temperatures fit or due to the fact that only one field was considered in the Curie–Weiss fits.

The role of superexchange in the magnetocaloric effect is examined in Figure 6c, which depicts the difference between the mean-field model predicted entropy change, Δ*S*_*m*,*Hext*+*J*1_, and the measured entropy change Δ*S*_*m*,*obs*_ for 2–20 K and 0–7 T. The mean-field model reduces the difference between the predicted and measured magnetic entropies to 1, 2, and 4% of *R* ln(2*S* + 1) for Ba_2_GdSbO_6_, Sr_2_GdSbO_6_, and Ca_2_GdSbO_6_, approximately 1, 2, and 5% of the max entropy observed. Furthermore, Figure S3 hows that the mean-field model accurately captures the saturation field and overall magnitude of (*∂M*/*∂T*)_*H*_ at low temperatures, to within the error of the data, compared to the free-spin prediction for both *fcc* compounds. The mean-field prediction for (*∂M*/*∂T*)_*H*_ of Ca_2_GdSbO_6_ at 2 K is not in as good of agreement with the measured data likely due to the presence of site disorder, onset of long-range order (*T*_0_ ∼ 0.52 K), or a larger dipolar contribution . Further experimental validation of these exchange constants could be accomplished by low temperature neutron magnetic diffuse scattering experiments to probe short- and long-range correlations between spins. At temperatures of 2 K and above, the mean-field model with antiferromagnetic superexchange thus serves as a good prediction of the observed magnetocaloric effect in *A*_2_GdSbO_6_.

## Comparison to Top Performing Gd^3+^ Magnetocaloric Materials

**Figure 7:**
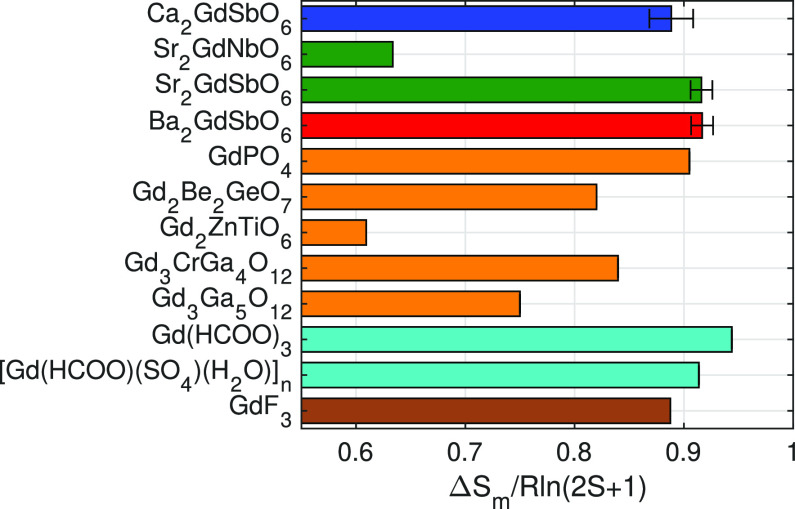
Magnetic entropy change Δ*S*_*m*_ of *A*_2_*Ln*SbO_6_ (*A* = {Ba, Sr, Ca}) in J/K/mol_Gd_ compared to top performing magnetocaloric materials from *H*_max_ = 7 T to zero field at 2 K, scaled by *R* ln(2*S* + 1).^3−5,27−31^ Dense lanthanide oxides are shown in orange, formate-based magnetocalorics in turquoise, ligand-based compounds in brown, and *fcc* lanthanide oxides in blue, green, and red. Note that the Gd_2_TiZnO_6_ value represented is at 2.1 K, as it occurs below the ordering transition. Error bars for Δ*S*_*m*_ of *A*_2_GdSbO_6_ were estimated using propagation of errors for a mass uncertainty of ±0.1 mg.

The magnetic entropy change Δ*S*_*m*_ attained by the *A*_2_GdSbO_6_ compounds at 2 K and a field of 7 T is compared to those of other top-performing Gd-based magnetocaloric materials in Figure 7. When comparing the entropy change per mole of Gd, the *fcc A*_2_GdSbO_6_ compounds outperform the top dense oxide magnetocaloric, GdPO_4_, exhibiting an entropy change of 0.92 ± 0.1*R* ln(2*S* + 1), only 0.02*R* ln(2*S* + 1) below Gd(HCOO)_3_.^3,4^ It should be noted that this order of performance would inevitably change when comparing Δ*S*_*m*_ per unit volume or per unit mass; however, evaluating Δ*S*_*m*_ per mole of Gd highlights the role of superexchange in the magnetocaloric effect. For example, the other top performers Gd(HCOO)_3_ and GdPO_4_ have been reported to behave like paramagnets down to 2 K with minimal antiferromagnetic correlations and GdF_3_ similarly with minimal ferromagnetic correlations (Table 3). Along with the mean-field and free-spin analysis in the preceding sections, these results suggest that minimal coupling between spins plays an important role in maximizing the magnetocaloric effect.

Table 3 lists the *nn* exchange constant *J*_1_ and dipolar interaction *D* in each of the materials estimated from the reported Curie–Weiss temperature Θ and crystal structure.

The top performing materials, Gd(HCOO)_3_, and GdF_3_ all have a *D*/*J*_1_ ratio on the order of 1–1.5, indicating that a small dipolar interaction may also improve magnetocaloric performance. Notably, *J*_1_ for the *fcc A*_2_GdSbO_6_ is around 15 mK or less, approximately 0.1–0.5 of the estimated *nn* exchange in the other materials shown and comparable to common paramagnetic salts, including FAA, CAS, and CPS.^35,39^ Aside from Gd_3_Ga_5_O_12_, Sr_2_GdSbO_6_ and Ba_2_GdSbO_6_ have the largest Debye temperatures, indicating the smallest lattice heat capacities, an ideal property in magnetocaloric applications.^3−5^

The *A*_2_GdSbO_6_ (*A*={Ba,Sr,Ca}) materials investigated in this work provide evidence that minimal superexchange is important in enhancing the magnetocaloric effect in lanthanide oxides. Furthermore, the frustrated *fcc* geometry of *A* = {Ba, Sr} and antiferromagnetic superexchange enable enhanced cooling to at least 400 mK in contrast to some nonfrustrated candidates such as GdF_3_ and Gd(HCOO)_3_, which are limited to their ordering temperatures of 1.25 and 0.8 K, respectively. Although Gd(HCOO)_3_ may exhibit a better magnetocaloric effect per unit volume or mass, the *fcc* double perovskite structure is more chemically tunable and thus allows for the temperature and magnitude of Δ*S*_*m*_ to be tuned. For example, one useful future study would be to investigate partial substitution of Sb^5+^ on the *B* sites or *A* site substitution. For Gd_3_Ga_5_O_12_, replacement of a single Ga^3+^ ion with Cr^3+^ improved the entropy change by over 10%.^30^

The role of the *M*^5+^ B site ion in the superexchange is highlighted by the recent report of the magnetocaloric effect in Sr_2_GdNbO_6_. Sr_2_GdNbO_6_ shows differing fundamental magnetic properties (i.e., ferromagnetic interactions), resembling *d*^0^ versus *d*^10^ distinctions observed in transition metal oxides.^42,43^ This material exhibits a maximum magnetocaloric effect near its ordering temperature (3 K) for μ_0_*H* = 7 T, −15.5 J/K/mol,^31^ comparable to the performance of Sr_2_GdSbO_6_ at 2 K reported in this work.

Our results indicate that changes in the magnetic lattice, such as site disorder in *A* = Ca, do not substantially alter the magnetocaloric effect for the *A*_2_GdSbO_6_ series at *T* ≥ 2 K. However, disorder does play a role in the magnetic ordering of the compounds, with *A* = Ca exhibiting a transition at 0.52 K and *A* = {Ba, Sr} remaining disordered down to 0.4 K. Future low-temperature heat capacity in applied field, μ-SR, and/or low-temperature neutron diffraction using isotopically enriched samples will be important in understanding how disorder affects the low temperature magnetic behavior. Disorder has recently been shown to play a role in the magnetocaloric effect observed in *A*GdS_2_, *A* = {Li, Na}, with a significant enhancement of the magnetocaloric effect observed in ordered NaGdS_2_ compared to cation disordered LiGdS_2_. This is rationalized by differences in the exchange interaction and the onset of ordering at higher temperatures in LiGdS_2_. At high temperatures, *T* > 2 K, a similar effect is not observed in the *A*_2_GdSbO_6_ double perovskites but may result in significant differences in the magnetocaloric effect closer to the ordering temperature in Ca_2_GdSbO_6_.^44^

The *fcc* materials presented here, Ba_2_GdSbO_6_ and Sr_2_GdSbO_6_, are likely able to cool below the industry standard, Gd_3_Ga_5_O_12_ (which has a lower cooling limit of  K, due to spin–spin correlations^45^), into the temperature regime of paramagnetic salts ( mK or less^46^) based on their minimal superexchange. This presents a possible significant advancement as the frustrating lattice should have a better per unit volume magnetic entropy change than a Gd^3+^-based paramagnetic salt.

## Conclusion

We synthesized three frustrated lanthanide oxides *A*_2_GdSbO_6_ (*A* = {Ba, Sr, Ca}) and characterized their structural and magnetic properties through X-ray powder diffraction and bulk magnetic measurements. The frustrated *fcc* lattice and small (*J*_1_ ∼ 10 mK) antiferromagnetic superexchange of Ba_2_GdSbO_6_ and Sr_2_GdSbO_6_ prevents magnetic ordering down to 0.4 K. In contrast, Ca_2_GdSbO_6_ is found to be site-disordered, with all Gd^3+^ ions lying on the *A* sites and an antiferromagnetic ordering transition at 0.52 K.

Intriguingly, all three materials make promising magnetocaloric candidates in the liquid He regime (2–20 K), achieving up to 92(1)% of the ideal magnetic entropy change *R* ln(2*S* + 1) in an applied field of up to 7 T. The comparable, high magnetocaloric performance (Δ*S*_*m*_ = 0.88(2)*R* ln(2*S* + 1)) of the site-disordered compound Ca_2_GdSbO_6_ suggests that the magnetocaloric effect is governed by primarily free-spin behavior at these temperatures. We demonstrate that the measured magnetocaloric effect of the frustrated Ba_2_GdSbO_6_ and Sr_2_GdSbO_6_ can be predicted to within experimental uncertainty using a mean-field model with a fit *nn* superexchange constant, *J*_1_. These results suggest that future top-performing Gd-based magnetocaloric materials should search for a balance between minimal superexchange between magnetic ions and frustration to suppress the magnetic ordering temperature. The tunability of the double perovskites via chemical substitution makes the *fcc* lanthanide oxides a promising set of materials for magnetic refrigeration.

## Supporting Information Available

Additional magnetic characterization, free Heisenberg spin model, and *M*(*H*) mean field exchange model (PDF)
