# Supplementary material for: Free-Spin Dominated Magnetocaloric Effect in Dense Gd3+ Double Perovskites
Source: Chem Mater. 2022 Mar 29;34(7):3440–50. doi: 10.1021/acs.chemmater.2c00261 (PMC9098179; doi:10.1021/acs.chemmater.2c00261)
Supplement: Supplementary file 1 — cm2c00261_si_001.pdf [file cm2c00261_si_001.pdf]

Supporting Information: Free-spin dominated magnetocaloric effect in dense Gd^{3+} double perovskites

EliseAnne C. Koskelo,[†,‡] Cheng Liu,[†] Paromita Mukherjee,[†] Nicola D. Kelly,[†] and Siân E. Dutton[*,†]

† *Department of Physics, University of Cambridge, Cambridge CB3 0HE, United Kingdom*

‡*Now at Department of Physics, Harvard University, Cambridge, MA 02138, USA.*

E-mail: sed33@cam.ac.uk

Magnetic Characterization

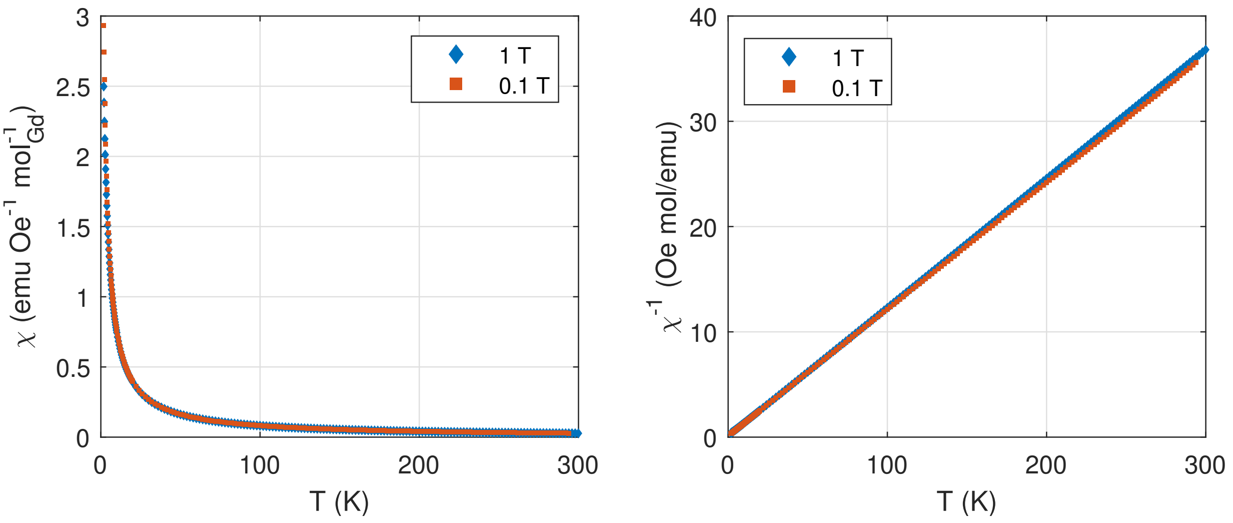

Figure S1: Zero-field-cooled magnetic susceptibility χ and of χ^{-1} Ca_2GdSbO_6 measured in a field of 1 T and 0.1 T. Error bars are smaller than points on graph.

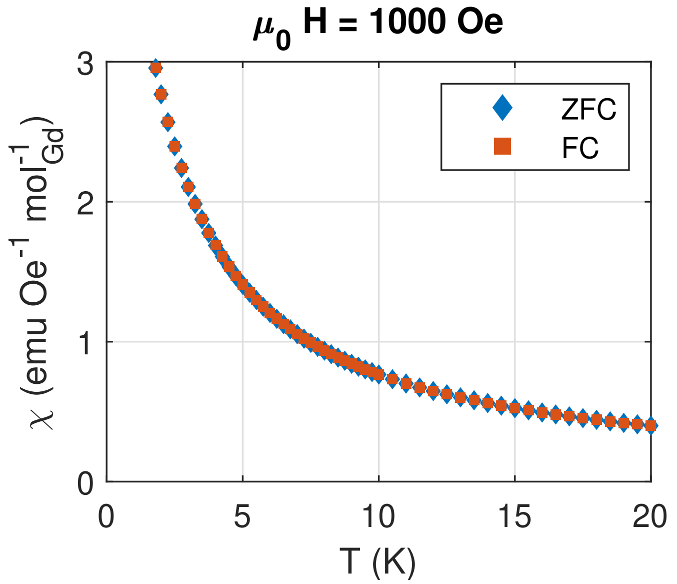

Figure S2: $\chi(T)$ of Ca_2GdSbO_6 measured in a field of 0.1 T under ZFC and FC conditions. Error bars are smaller than points on graph.

Curie-Weiss Superexchange and Dipolar Estimates

For either antiferromagnetic or ferromagnetic exchange, the mean-field approximation enables an estimation of the nn exchange energy J_1 from the Curie-Weiss temperature, Θ, via:[1]

$$J_1 = \frac{-3\Theta}{zS(S+1)} \tag{S1}$$

where z is the number of nearest neighbors for a single magnetic ion with total angular momentum J (e.g. $z = 12$ for the *fcc* lattice). The dipole-dipole interaction between magnetic ions can be estimated using:

$$D_{nn} = \frac{\mu_0 \mu_{eff}^2}{4\pi R_{nn}^3 k_B}, \tag{S2}$$

where $\mu_{eff} = g_J\sqrt{J(J+1)}$ is the effective magnetic moment (equivalent to $g_J\sqrt{S(S+1)}$ for Gd^{3+}, Fe^{3+}, and Cu^{2+}) and R_{nn} is the nn distance between ions.[1]

Free Heisenberg Spins Model

For an applied field $\mu_0 H$ at temperature T, the bulk magnetization M_S of the system of uncoupled spins S is given by:

$$M_S = M_{sat} B_J \left(\frac{g_J \mu_B J \mu_0 H}{k_B T} \right), \tag{S3}$$

where $M_{sat} = g_J J \mu_B$ is the saturation magnetization per unit spin and

$$B_J(y) = \frac{2J+1}{2J} \coth\left(\frac{2J+1}{2J} y\right) - \frac{1}{2J} \coth\left(\frac{y}{2J}\right), \tag{S4}$$

is the Brillouin function.

$M(H)$-Based Mean-Field Exchange Model

As described in the text, a global fit to the measured isothermal magnetization $M(H)$ using Equation 8 was used to determine the nn superexchange J_1 for both Ba_2GdSbO_6 and Sr_2GdSbO_6 and an overall exchange field, $a_{ex} \propto -J_1$ for the site-disordered $[CaGd]_A[CaSb]_BO_6$. The resulting fits are depicted in Figure 6 for temperatures of 2, 4, 6, 8, 10, and 15 K. In all cases the maximum magnetization was scaled by 1.04 in agreement with the observed value, $1.04g_JJ$, as in Reference 2.

Model predictions for the temperature gradient of the magnetization $(\partial M/\partial T)_H$ (solid lines) are compared to the observed data (points) in Figure S3. Dotted lines correspond to the prediction for uncoupled Heisenberg spins.

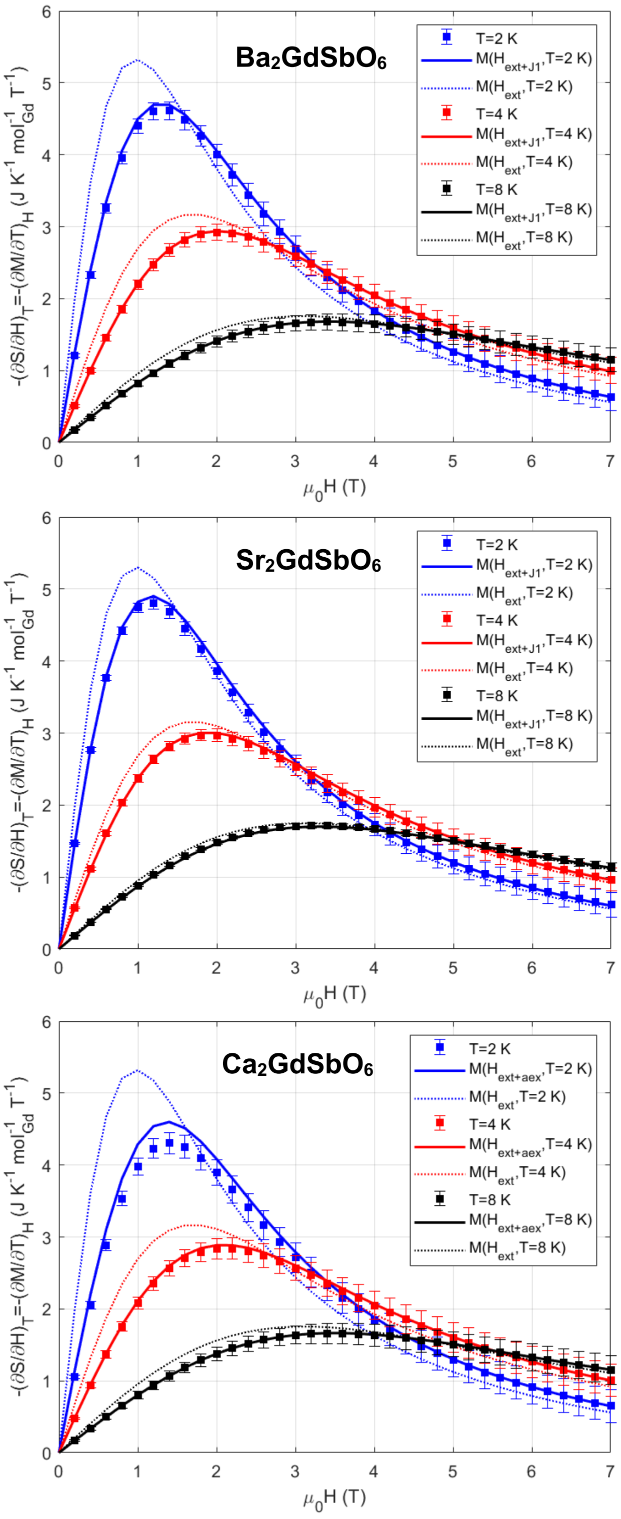

Figure S3: Temperature derivative of the magnetization $(\partial M/\partial T)_H$ predicted for free Heisenberg $S = 7/2$ spins in an external field M_{Hext} (dotted lines) and in the nn exchange field model using the fit J_1, $M_{Hext+J1}$ (solid lines) compared to experimental measurements (data points). Error bars for $(\partial M/\partial T)_H$ are determined assuming a mass error of ± 0.1 mg.

References

(1) Paddison, J.; Ehlers, G.; Petrenko, O.; Wildes, A.; Gardner, J.; Stewart, J. Spin correlations in the dipolar pyrochlore antiferromagnet $Gd_2Sn_2O_7$. *Journal of Physics: Condensed Matter* **2017**, *29*, 144001.

(2) Wellm, C.; Zeisner, J.; Alfonsov, A.; Sturza, M.-I.; Bastien, G.; Gaß, S.; Wurmehl, S.; Wolter, A. U. B.; Büchner, B.; Kataev, V. Magnetic interactions in the tripod kagome antiferromagnet $Mg_2Gd_3Sb_3O_{14}$ probed by static magnetometry and high-field ESR spectroscopy. *Phys. Rev. B* **2020**, *102*, 214414.
